# Supplementary material for: Wastewater-based epidemiology to enhance public health preparedness and response during large-scale events: experiences from the 2024 Republican and Democratic National Conventions – Milwaukee, WI and Chicago, IL
Source: Front Public Health. 2026 Mar 19;14:1800385. doi: 10.3389/fpubh.2026.1800385 (PMC13044080; doi:10.3389/fpubh.2026.1800385)
Supplement: Supplementary file 1 [file Supplementary_file_1.docx]

**Wastewater-based epidemiology to enhance public health preparedness and response during large-scale events: experience from the 2024 Republican and Democratic National Conventions – Milwaukee, WI, and Chicago, IL**

**Supplementary information:**

**UWM concentration and extraction details:**

A description of the HA filtration is available on protocols.io [dx.doi.org/10.17504/protocols.io.bxu7pnzn](https://dx.doi.org/10.17504/protocols.io.bxu7pnzn). Prior to the total nucleic acid (TNA) extraction with the NucleoMag® DNA/RNA Water Kit, samples were bead-bashed for two cycles of 150 seconds using a mini bead beater (Biospec Products) before being spun down. A total of 400μL was extracted and eluted into 100μL with DNA/RNA-free water. More details about the extraction are available on protocols.io [**dx.doi.org/10.17504/protocols.io.q26g7yjq9gwz/v2**](https://dx.doi.org/10.17504/protocols.io.q26g7yjq9gwz/v2).

**WSLH concentration and extraction** **details:**

A description of the Ceres Nanotrap concentration is available on protocols.io [dx.doi.org/10.17504/protocols.io.261gedz7dv47/v1](https://dx.doi.org/10.17504/protocols.io.261gedz7dv47/v1). Nucleic acids were extracted with the Maxwell® HT Environmental TNA kit (Promega, Cat# AX9190) on a KingFisher Flex (Thermo Fisher Scientific) according to manufacturer instructions. Briefly, half of the nanotrap-concentrated eluate was used in TNA extraction/purification. Once purified, the total nucleic acid was eluted in 200μL of 25mM Tris-HCl. More details about the extraction are available on protocols.io [dx.doi.org/10.17504/protocols.io.rm7vzx4wxgx1/v1](https://dx.doi.org/10.17504/protocols.io.rm7vzx4wxgx1/v1).

To quantify Hepatitis A, 50mL of raw wastewater was centrifuged at 3950 rpm (approximately equivalent of 4000xg) for 12 min. After discarding the supernatant, pellets were resuspended into 400μL of CTAB lysis buffer (Promega, Cat# MC1411) before being transferred into Lysing Matrix A tubes (MP Biomedicals). Tubes were bead-bashed for 90 seconds at 7m/sec using the FastPrep-24™ 5G system (MP Biomedicals). After spinning down the bead-bashed samples at 10,000 rpm (equivalent of 9170xg) for 2 min at 4°C, 250μL was extracted using the Maxwell® HT Environmental TNA kit (Promega), as described above.

**Viral and bacterial quantification details:**

UWM performed all their quantifications using the Bio-Rad QX200 Droplet Digital PCR System. WSLH and UIC used the QIAGEN QIAcuity Digital PCR System. Additional information about the assays, PCR reagents, and conditions is listed in Tables S1 and S2. All assays included a no template control (NTC) of nuclease-free water, and a quantitative positive control. The UWM and WSLH quantification protocols are available on protocols.io ([dx.doi.org/10.17504/protocols.io.81wgbwx5ygpk/v1](https://dx.doi.org/10.17504/protocols.io.81wgbwx5ygpk/v1) and [dx.doi.org/10.17504/protocols.io.yxmvmex65g3p/v1](https://dx.doi.org/10.17504/protocols.io.yxmvmex65g3p/v1)).

At the UWM, an inhibition assay using bovine respiratory syncytial virus (BRSV) was assessed on all samples as described previously^1^. None of the samples used in this study exhibited any signs of inhibition. Sample blanks were carried out through filtration and extraction biweekly to confirm there was no contamination present. The inhibition was also evaluated using BRSV assay at the WSLH, but only on the extracts from centrifugation method (for Hepatitis A assessment). UIC did not assess the inhibition.

At the UWM, the limit of detection (LOD) was defined as the lowest analyte concentration likely to be reliably distinguished from the limit of blank (LOB) and at which the detection is feasible. The LOB was calculated as the maximum value from a 90% confidence interval of the average false positive droplets across 24 replicate measurements (see Feng et al. 2021^1^ for more details). At WSLH, the LOB was calculated as the maximum number of positive partitions observed across a minimum of 23 NTCs. The LOD was defined as the lowest concentration (of the target positive control) that is consistently detected in at least 95% of the replicates. For more details about the LOD determination see https://doi.org/10.6084/m9.figshare.24467917. At the UIC, a serial dilution of a control was performed and the LOD was defined as the dilution at which a single positive partition was observed.

LOB and LOD values are described in Tables S1 and S2.

ddPCR raw droplet amplification data were initially analyzed and extracted from the Bio-Rad QuantaSoft Analysis software and processed using R package twoddpcr (version 1.30.0).^2^
dPCR data were analyzed using the QIAcuity Software Suite 2.5.0.

Table S1 Description of the PCR assays for RNA targets

|  |  |  |  |  |  |  |  |  |  |  | **Thermocycling Parameters** | | | | | |
| --- | --- | --- | --- | --- | --- | --- | --- | --- | --- | --- | --- | --- | --- | --- | --- | --- |
| **Assay** | | **Sequence 5’-3’^†^** | **Source** | **Laboratory** | **Platform^‡^** | **Final concentration^ꬸ^** | **Volume template added** | **Positive control used^ꬹ^** | **Limit of blank** | **Limit of detection** | **Reverse transcription** | **Activation** | **40 cycles** | | **Deactivation for ddPCR only** | **Stabilization for ddPCR only** |
|  |  |  |  |  |  |  |  |  |  |  |  |  | **Denaturation** | **Annealing** |  |  |
| SARS-CoV-2 | N1 | F: GACCCCAAAATCAGCGAAAT | 3 | UWM  *N1 and N2* | ddPCR | F/R: 900 nM P: 250 nM | 5 uL of RNA diluted up to 1:3 | Exact Diagnostics SARS-CoV-2 standards (Bio-Rad) | 0 positive droplet | 3 positive droplets for N1 and N2 | 60 min  50℃ | 10 min  95℃ | 30 sec  94℃ | 1 min  55℃ | 10 min  98℃ | 30 min and hold 4℃ |
|  |  | R: TCTGGTTACTGCCAGTTGAATCTG |  |  |  |  |  |  |  |  |  |  |  |  |  |  |
|  |  | P: FAM/ACCCCGCAT/ZEN/TACGTTTGGTGGACC/IABkFQ |  |  |  |  |  |  |  |  |  |  |  |  |  |  |
|  | N2 | F: TTACAAACATTGGCCGCAAA |  |  |  |  |  |  |  |  |  |  |  |  |  |  |
|  |  | R: GCGCGACATTCCGAAGAA |  |  |  |  |  |  |  |  |  |  |  |  |  |  |
|  |  | P: HEX/ACAATTTGC/ZEN/CCCCAGCGCTTCAG/IABkFQ |  |  |  |  |  |  |  |  |  |  |  |  |  |  |
| Influenza A | | F1: CAAGACCAATCYTGTCACCTCTGAC | 4 | WSLH  *Influenza A, Influenza B, RSV* | dPCR  *24-well plates* | F1/F2: 500 nM  R1: 750 nM  R2: 250 nM,  P: 250 nM | 5 uL of undiluted RNA | Clinical RNA strain and ultramer^a^ | 0 positive partition | 3 positive partitions (~50000 cp/L) | 30 min  50℃ | 2 min  95℃ | 10 sec  95℃ | 30 sec  55℃ | - | - |
|  |  | F2: CAAGACCAATYCTGTCACCTYTGAC |  |  |  |  |  |  |  |  |  |  |  |  |  |  |
|  |  | R1: GCATTYTGGACAAAVCGTCTACG |  |  |  |  |  |  |  |  |  |  |  |  |  |  |
|  |  | R2: GCATTTTGGATAAAGCGTCTACG |  |  |  |  |  |  |  |  |  |  |  |  |  |  |
|  |  | P: TGCAGTCCTCGCTCACTGGGCACG (FAM/ZEN/3IABkFQ) |  |  |  |  |  |  |  |  |  |  |  |  |  |  |
| Influenza B | | F: TCCTCAAYTCACTCTTCGAGCG |  |  |  | F/R: 1000 nM  P: 250 nM |  | Clinical RNA strain and ultramer^b^ | 0 positive partition | 3 positive partitions (~50000 cp/L) |  |  |  |  |  |  |
|  |  | R: CGGTGCTCTTGACCAAATTGG |  |  |  |  |  |  |  |  |  |  |  |  |  |  |
|  |  | P: HEX/CCAATTCGA/ZEN/GCAGCTGAAACTGCGGTG/3IABkFQ |  |  |  |  |  |  |  |  |  |  |  |  |  |  |
| Respiratory Syncytial Virus | | CTCCAGAATAYAGGCATGAYTCTCC | 5 |  |  | F/R: 900 nM  P: 250 nM |  | Clinical RNA strain and ultramer^c^ | 0 positive partition | 3 positive partitions (~50000 cp/L) |  |  |  |  |  |  |
|  |  | R: GCYCTYCTAATYACWGCTGTAAGAC |  |  |  |  |  |  |  |  |  |  |  |  |  |  |
|  |  | P: TAMN/TAACCAAATTAGCAGCAGGAGATAGATCAG/3IAbRQSp |  |  |  |  |  |  |  |  |  |  |  |  |  |  |
| SARS-CoV-2 | | GT-Digital Flu;SC2;RSV Wastewater Surveillance Panel for the QIAcuity™ Digital PCR System | - | UIC  *SARS-CoV-2,*  *Influenza A, Influenza B, RSV* | dPCR  *24-well plates* | 1X | 5 uL of RNA diluted up to 1:1 | Included in the kit | 0 positive partitions | 1 positive partition (~2100 cp/L) | 30 min  50℃ | 2 min  95℃ | 10 sec  95℃ | 30 sec  55℃ | - | - |
| Influenza A | |  |  |  |  |  |  |  |  |  |  |  |  |  |  |  |
| Influenza B | |  |  |  |  |  |  |  |  |  |  |  |  |  |  |  |
| Respiratory Syncytial Virus | |  |  |  |  |  |  |  |  |  |  |  |  |  |  |  |
| Norovirus | GI | F: CGYTGGATGCGITTYCATGA | 6 | WSLH  *GI and GII* | dPCR  *96-well plates* | F/R: 400 nM  P: 200 nM | 6 uL of 1:4 RNA | Ultramer^d^ | 0 positive partition | 5 positive partitions (~600000 cp/L) | 40 min  50℃ | 2 min  95℃ | 15 sec  95℃ | 30 sec  60℃ | - | - |
|  |  | R: CTTAGACGCCATCATCATTYAC |  |  |  |  |  |  |  |  |  |  |  |  |  |  |
|  |  | P: HEX/TGG+AC+AG+GRG+AYCGC/3IABkFQ |  |  |  |  |  |  |  |  |  |  |  |  |  |  |
|  | GII | F: CARGARBCNATGTTYAGRTGGATGAG |  |  |  |  |  | Ultramer^e^ | 0 positive partition | 5 positive partitions (~600000 cp/L) |  |  |  |  |  |  |
|  |  | R: TCGACGCCATCTTCATTCACA |  |  |  |  |  |  |  |  |  |  |  |  |  |  |
|  |  | P: FAM/TGGGAGGGC/ZEN/GATCGCAATCT/3IABkFQ |  |  |  |  |  |  |  |  |  |  |  |  |  |  |
| Norovirus genotype I and II | | GT-Digital Norovirus Wastewater Surveillance Panel for the QIAcuity™ Digital PCR System | - | UIC  *GI and GII* | dPCR  *24-well plates* | 1X | 5 uL of RNA diluted up to 1:1 | Included in the kit | 0 positive partitions | 1 positive partition (~1900 cp/L) | 30 min  50℃ | 2 min  95℃ | 10 sec  95℃ | 30 sec  55℃ | - | - |
| Measles | | F: TGGCATCTGAACTCGGTATCAC | 7 | WSLH  *Measles, Mumps, Rubella* | dPCR  *24-well plates* | F/R: 400 nM  P: 200 nM | 5 uL of undiluted RNA | Clinical RNA | 0 positive partition | 3 positive partitions (~50000 cp/L) | 40 min  50℃ | 2 min  95℃ | 15 sec  95℃ | 60 sec  58℃ | - | - |
|  |  | R: TGTCCTCAGTAGTATGCATTGCAA |  |  |  |  |  |  |  |  |  |  |  |  |  |  |
|  |  | P: FAM/CCGAGGATG/ZEN/CAAGGCTTGTTTCAGA/3IABkFQ |  |  |  |  |  |  |  |  |  |  |  |  |  |  |
| Mumps | | F: GTATGACAGCDTACGACCAACCT | 8 |  |  | F/R: 400 nM  P: 200 nM |  |  | 0 positive partition | 3 positive partitions (~50000 cp/L) |  |  |  |  |  |  |
|  |  | R: GCGACCTTGCTGCTGGTATT |  |  |  |  |  |  |  |  |  |  |  |  |  |  |
|  |  | P: SUN/CYGGRTCTG/ZEN/CTGATCGGCGAT/3IABkFQ |  |  |  |  |  |  |  |  |  |  |  |  |  |  |
| Rubella | | F: CAGATGCAGGTTAGTGATCA | 9 |  |  | F/R: 900 nM  P: 100 nM |  |  | 0 positive partition | 3 positive partitions (~50000 cp/L) |  |  |  |  |  |  |
|  |  | R: GACGTGTAGGGCTTCTTTAG |  |  |  |  |  |  |  |  |  |  |  |  |  |  |
|  |  | P: Cy5/CCCGCCGCC/TAO/ATTGGATCGAG/3IAbRQSp |  |  |  |  |  |  |  |  |  |  |  |  |  |  |
| Hepatitis A | | F: GTAACAGCGGCGGATATTGG | 10 | WSLH  *Hepatitis A* | dPCR  *24-well plates* | F/R: 400 nM  P: 200 nM | 5 uL of undiluted RNA | Ultramer^f^ | 0 positive partition | 3 positive partitions (~8000 cp/L) | 40 min  50℃ | 2 min  95℃ | 15 sec 95℃ | 30 sec 60℃ | - | - |
|  |  | R: CCTAGAGACAGCCCTGACA |  |  |  |  |  |  |  |  |  |  |  |  |  |  |
|  |  | P: FAM/CAACGCCGG/ZEN/AGGACTGGCTCTCATCCAG/3IABkFQ |  |  |  |  |  |  |  |  |  |  |  |  |  |  |

ⴕUWM: Primers/probes ordered from Integrated DNA Technologies (IDT). WSLH: Probes ordered from IDT and primers ordered either from Invitrogen or IDT.
‡ddPCR: Bio-Rad QX200 Droplet Digital PCR System; dPCR: QIAGEN QIAcuity Digital PCR System run using QIAcuity 24-well plates (40 uL total Rx volume, up to 26000 partitions per Rx) or QIAcuity 96-well plates (12 uL total Rx volume, up to 8500 partitions per Rx)
ꬸUWM: One-Step RT-ddPCR Advanced Kit for Probes and ddPCR Supermix. WSLH: QIAcuity One-Step ADVANCED RT-PCR mastermix and QuantiNova Reverse Transcriptase (Qiagen). UIC: QIAcuity OneStepAdvanced Probe Kit. All at a final concentration of 1X.

ꬹUltramers and gBlocks ordered from IDT. Positive control sequences:

^a^AAGACAAGACCAATTCTGTCACCTTTGACTAAGGGGATTTTAGGGTTTGTTTTCACGCTCACCGTGCCCAGTGAGCGAGGACTGCAGCGTAGACGCTTTGTCCAAAATGCCCTCAATGGGAATGG (OP872047.1)

^b^TCCTCAACTCACTCTTCGAGCGTCTCAATGAAGGACATTCAAAGCCAATTCGAGCAGCTGAAACTGCGGTGGGAGTCTTATCCCAATTTGGTCAAGAGCACCG (OR052893.1)

^c^CTCCAGAATATAGGCATGATTCTCCAGACTGTGGGATGATAATACTGTGTATGGCTGCTCTTGTAATAACCAAATTAGCAGCAGGAGATAGATCAGGTCTTACAGCAGTAATTAGGAGGGC (LC741419.1)

^d^CGCTGGATGCGATTCCATGATTTGAGCTTGTGGACAGGAGACCGCGATCTCTTGCCCGATTATGTAAATGATGATGGCGTCTAAG

^e^CAAGAGTCAATGTTCAGATGGATGAGGTTCTCAGATCTAAGCACATGGGAGGGCGATCGCAATCTGGCTCCCAGTTTTGTGAATGAAGATGGCGTCGA

^l^TTCTATGAAGAGATGCTTTGGATAGGGTAACAGCGGCGGATATTGGTGAGTTGTTTGACAAAAACCATTCAACGCCGGAGGACTGGCTCTCATCCAGTGGATGCATTAAGTGGATTGTCTGTCAGGGCTGTCTCTAGGTTTAATTCCTGACCTCTCTGTGCT

Table S2 Description of the PCR assays for DNA targets

|  |  |  |  |  |  |  |  |  |  |  | **Thermocycling Parameters** | | | | | |
| --- | --- | --- | --- | --- | --- | --- | --- | --- | --- | --- | --- | --- | --- | --- | --- | --- |
| **Assay** | | **Sequence 5’-3’^†^** | **Source** | **Laboratory** | **Platform^‡^** | **Final concentration^ꬸ^** | **Volume template added** | **Positive control used^ꬹ^** | **Limit of blank** | **Limit of detection** | **Activation** | **40 cycles** | | | **Deactivation for ddPCR only** | **Stabilization for ddPCR only** |
|  |  |  |  |  |  |  |  |  |  |  |  | **Denaturation** | **Annealing** | **Extension** |  |  |
| *Salmonella* (invA gene) | | F: TCGTCATTCCATTACCTACC | 11 | UWM | ddPCR | F/R: 900 nM P: 250 nM | 5 uL of RNA diluted up to 1:3 | gBlock^f^ | 0 positive droplet | 1 positive droplet (~ 0.075219725 cp/ul Rx) | 10 min  95℃ | 30 sec  94℃ | 1 min  53℃ | - | 10 min  98℃ | 30 min to infinite 4℃ |
|  |  | R: AAACGTTGAAAAACTGAGGA |  |  |  |  |  |  |  |  |  |  |  |  |  |  |
|  |  |  |  | UIC | dPCR  24-well plates | F/R: 800 nM P: 400 nM | 5 uL of RNA diluted up to 1:1 | gBlock^g^ | 0 positive partition | 1 positive partition (~2100 cp/L) | 2 min  95℃ | 10 sec  95℃ | 30 sec  48℃ | 2 min  55℃ | - | - |
|  |  | P: FAM/TCTGGTTGA/ZEN/TTTCCTGATCGCA/IABkFQ |  |  |  |  |  |  |  |  |  |  |  |  |  |  |
| Shiga toxin1-producing bacteria | Stx1 | F: ACATTGTCTGGTGACAGTAGC | 12 | UWM  *Stx1 and Stx2* | ddPCR | F/R: 900 nM P: 250 nM | 5 uL of DNA diluted up to 1:3 | gBlocks: Stx1^h^, Stx2^i^ | 0 positive droplet | 1 positive droplet (~ 0.075219725 cp/ul Rx) for Stx1 and Stx2 | 10 min  95℃ | 30 sec  94℃ | 1 min  60℃ | - | 10 min  98℃ | 30 min to infinite 4℃ |
|  |  | R: CGACATTAAATCCAGATAAGAAGTAGT |  |  |  |  |  |  |  |  |  |  |  |  |  |  |
|  |  | P: FAM/ATCAGTCGT/ZEN/ACGGGGATGCAGATAAAT/IABkFQ |  |  |  |  |  |  |  |  |  |  |  |  |  |  |
|  | Stx2 | F: ATGACAACGGACAGCAGTTAT |  | UIC  *Stx1 and Stx2* | dPCR  24-well plates | F/R: 800 nM P: 400 nM | 5 uL of RNA diluted up to 1:1 | gBlocks: Stx1^j^, Stx2^k^ | 0 positive partition | Stx1: 1 positive partition (~2000 cp/L), Stx2: 1 positive partition (~4000 cp/L) | 2 min  95℃ | 10 sec 95℃ | 30 sec  51℃ | 2 min  56℃ | - | - |
|  |  | R: CTGAACTCCATTAACGCCAGATA |  |  |  |  |  |  |  |  |  |  |  |  |  |  |
|  |  | P: HEX/ATGCAAATC/ZEN/AGTCGTCACTCACTGG/IABkFQ |  |  |  |  |  |  |  |  |  |  |  |  |  |  |

**†**UWM/UIC: Primers/probes ordered from Integrated DNA Technologies (IDT). WSLH: Probes ordered from IDT and primers ordered either from Invitrogen or IDT.
‡ddPCR: Bio-Rad QX200 Droplet Digital PCR System; dPCR: QIAGEN QIAcuity Digital PCR System run using QIAcuity 24-well plates (40 uL total Rx volume, up to 26000 partitions per Rx) or QIAcuity 96-well plates (12 uL total Rx volume, up to 8500 partitions per Rx)
ꬸUWM: ddPCR Supermix for Probes (No dUTP). UIC: Qiagen QIAcuity Probe PCR Kit. Both at a 1X final concentration.

ꬹUltramers and gBlocks ordered from IDT. Positive control sequences:

^a^ TCTATGTTCGTCATTCCATTACCTACCTATCTGGTTGATTTCCTGATCGCACTGAATATCGTACTGGCGATATTGGTGTTTATGGGGTCGTTCTACATTGACAGAATCCTCAGTTTTTCAACGTTTCCTGCG

^b^TCTATGTTCGTCATTCCATTACCTACCTATCTGGTTGATTTCCTGATCGCACTGAATATCGTACTGGCGATATTGGTGTTTATGGGGTCGTTCTACATTGACAGAATCCTCAGTTTTTCAACGTTTCCTGCG

^c^TCGGTTACATTGTCTGGTGACAGTAGCGTTACAGTATACCACTGCAGGGCGTGTATCAGTCGTACGGGGATGCAGATAAATCTCGTTGGCCATACTACTTCTTATCTGGATTTAATGTCGCATAGA

^d^CGTTATTGAATGACAACGGACAGCAGTTATGTCGCAGCGCTGGAACCACTCTGCAACGTACGTTCCGGAATGCAAATCAGTCGTCACTCACTGGATCATTTCTATCTGGCGTTAATGGAGTTCAGCTGGACT

^e^TCGGTTACATTGTCTGGTGACAGTAGCGTTACAGTATACCACTGCAGGGCGTGTATCAGTCGTACGGGGATGCAGATAAATCTCGTTGGCCATACTACTTCTTATCTGGATTTAATGTCGCATAGA

^f^CGTTATTGAATGACAACGGACAGCAGTTATGTCGCAGCGCTGGAACCACTCTGCAACGTACGTTCCGGAATGCAAATCAGTCGTCACTCACTGGATCATTTCTATCTGGCGTTAATGGAGTTCAGCTGGACT

**Molecular sequencing and culturing at RIPHL:**

100μL of concentrated wastewater was inoculated to 9mL of Selenite broth with Cystine (ThermoFisher, R064508) and incubated at 35°C for 18-24 hours. 100μl broth was inoculated to HardyCHROM Salmonella agar (Hardy Diagnostics, G309) and streaked for isolation. All pink to magenta colonies were subcultured to blood agar and incubated at 35°C for 18-24 hours. MALDI-TOF MS was used to confirm *Salmonella* species. Genomic DNA was extracted from all *Salmonella* isolates, and sequencing libraries were prepared using the Nextera XT DNA Library Prep kit (Illumina, FC-131-1096). Libraries were sequenced using 2x150 bp reads on the NovaSeqX platform (Illumina). Raw reads were trimmed, filtered, assembled, and analyzed for MLST and serotype using the TheiaProk pipeline.^13^ Genomes were confirmed to be high quality (<200 contigs, >100x genome coverage, and genome size within 20% of 4.8 Mbp) and core SNP analysis was conducted using the Snippy Streamline pipeline.^13^  Raw sequence data was uploaded to NCBI Sequence Read Archive (SRA), SRR31174240- SRR31174244.

**Work cited**

1. Feng S, Roguet A, McClary-Gutierrez JS, et al., Evaluation of Sampling, Analysis, and Normalization Methods for SARS-CoV-2 Concentrations in Wastewater to Assess COVID-19 Burdens in Wisconsin Communities. *ACS EST Water*. 2021. 1(8):1955–65. doi: 10.1021/acsestwater.1c00160

2. Chiu A, Ayub M, Dive C, et al. twoddpcr: An R/Bioconductor Package and Shiny App for Droplet Digital PCR Analysis. *Bioinformatics*. 2017. 33(17):2743–45. doi: 10.1093/bioinformatics/btx308

3. CDC. 2019-Novel Coronavirus (2019-NCoV) Real-Time RRT-PCR Panel Primers and Probes Note. 2020. https://www.cdc.gov/coronavirus/2019-ncov/downloads/rt-pcr-panel-primer-probes.pdf (accessed 2021-06-07)

4. CDC. Research Use Only CDC Influenza SARS-CoV-2 (Flu SC2) Multiplex, <https://archive.cdc.gov/#/details?url=https://www.cdc.gov/coronavirus/2019-ncov/downloads/lab/multiplex-primers-probes-printer.pdf>, 2021 (accessed August 20, 2025)

5. Hughes B, Duong D, White BJ, et al. Respiratory Syncytial Virus (RSV) RNA in Wastewater Settled Solids Reflects RSV Clinical Positivity Rates. *Environ Sci Technol Lett*. 2022. 9(2):173–78. doi: 10.1021/acs.estlett.1c00963

6. Cannon JL, Barclay L, Collins NR, et al. Genetic and Epidemiologic Trends of Norovirus Outbreaks in the United States from 2013 to 2016 Demonstrated Emergence of Novel GII.4 Recombinant Viruses. *J Clin Microbiol*. 2017. 55(7):2208–21.
doi:10.1128/JCM.00455-17

7. Hummel KB, Lowe L, Bellini WJ, Rota PA. Development of QuantitativeGene-Specific Real-Time RT-PCR Assays for the detection of measles virus in clinical specimens. *J Virol Methods.* 2006. 132(1–2):166–73. doi:10.1016/j.jviromet.2005.10.006

8. Bryant P, Caldwell H, Lamson DM, et al. Streamlined Whole-Genome Sequencing of Mumps Virus for High-Resolution Outbreak Analysis. *J Clin Microbiol*. 2022. 60(1):e0084121. doi:10.1128/JCM.00841-21

9. Wu J, Wang MX, Kalvapalle P, et al. Multiplexed Detection, Partitioning, and Persistence of Wild-Type and Vaccine Strains of Measles, Mumps, and Rubella Viruses in Wastewater. *Environ Sci Technol.* 2024. 58(50):21930–41. doi:10.1021/acs.est.4c05344

10. Chou KX and Williams-Hill DM. Improved TaqMan real-time assays for detecting hepatitis A virus. *J Virol Methods.* 2018. 1;254:46–50. doi: 10.1016/j.jviromet.2018.01.014

11. Hoorfar J, Ahrens P, Rådström P. Automated 5′ Nuclease PCR Assay for Identification of *Salmonella enterica*. *J Clin Microbiol.* 2000. 38(9):3429-3435. doi:10.1128/jcm.38.9.3429-3435.2000

12. Derzelle S, Grine A, Madic J, et al. A Quantitative PCR Assay for the Detection and Quantification of Shiga Toxin-Producing *Escherichia coli* (STEC) in Minced Beef and Dairy Products. *Int J Food Microbiol.* 2011. 151(1):44-51. doi:10.1016/j.ijfoodmicro.2011.07.039

13. Libuit KG, Doughty EL, Otieno JR, et al. Accelerating bioinformatics implementation in public health. *Microb Genomics.* 2023. 9(7):mgen001051. doi:10.1099/mgen.0.001051
